# HGF/c-MET Axis in Tumor Microenvironment and Metastasis Formation

**DOI:** 10.3390/biomedicines3010071

**Published:** 2015-01-22

**Authors:** Anna Spina, Valeria De Pasquale, Giuliana Cerulo, Pasquale Cocchiaro, Rossella Della Morte, Luigi Avallone, Luigi Michele Pavone

**Affiliations:** 1 Department of Molecular Medicine and Medical Biotechnology, University of Naples Federico II, Via S. Pansini 5, 80131 Naples, Italy; E-Mails: annaspina82@gmail.com (A.S.); valeria.depasquale@unina.it (V.P.); cerulo@libero.it (G.C.); cocchiaropasquale@libero.it (P.C.); 2 Department of Veterinary Medicine and Animal Productions, University of Naples Federico II, Via F. Delpino 1, 80137 Naples, Italy; E-Mails: rosdella@unina.it (R.D.M.); luigi.avallone@unina.it (L.A.)

**Keywords:** HGF, c-MET, cancer, microenvironment, metastasis

## Abstract

Tumor metastases are responsible for approximately 90% of all cancer-related deaths. Metastasis formation is a multistep process that requires acquisition by tumor cells of a malignant phenotype that allows them to escape from the primary tumor site and invade other organs. Each step of this mechanism involves a deep crosstalk between tumor cells and their microenvironment where the host cells play a key role in influencing metastatic behavior through the release of many secreted factors. Among these signaling molecules, Hepatocyte Growth Factor (HGF) is released by many cell types of the tumor microenvironment to target its receptor c-MET within the cells of the primary tumor. Many studies reveal that HGF/c-MET axis is implicated in various human cancers, and genetic and epigenetic gain of functions of this signaling contributes to cancer development through a variety of mechanisms. In this review, we describe the specific types of cells in the tumor microenvironment that release HGF in order to promote the metastatic outgrowth through the activation of extracellular matrix remodeling, inflammation, migration, angiogenesis, and invasion. We dissect the potential use of new molecules that interfere with the HGF/c-MET axis as therapeutic targets for future clinical trials in cancer disease.

## 1. Introduction

Metastasis formation is an extremely complex, multistep, and multifunctional biological event that results from a complex molecular cascade through which cancer cells leave the site of the primary tumor and disseminate to distant organs, where they can proliferate and form secondary tumor foci. Metastasis arises through a series of adhesive interactions and invasive processes, as well as responses to chemotactic stimuli [1]. This cascade of events includes the development of new blood vessels, the “escape” of tumor cells from the primary tumor and their migration through the vessel basement membrane and extracellular matrix (ECM) surrounding the tumor epithelium, the invasion and the intravasation by the tumor cells of the endothelial basement membrane of local blood and/or lymphatic vessels, the adhesion of the circulating tumor cells to the endothelium of capillaries of the target organ site, the extravasation of the tumor cells through the endothelial cell layer and the surrounding basement membrane, and, finally, the growth of a secondary tumor [2].

The metastatic capabilities of cancer cells not only depend on cell-autonomous genetic and epigenetic alterations, but also on changes and adaptation due to the tumor microenvironment which is composed by a large variety of cell types, including fibroblasts, resident epithelial cells, pericytes, myofibroblasts, vascular and lymphovascular endothelial cells, and infiltrating cells of the immune system [3,4,5]. Together with the ECM, these non-malignant cell types constitute the stromal tissue of the tumor. The stromal cells nearby the growing tumor secrete ECM components, cytokines, and growth factors, thus enhancing tumor growth and invasion [6,7,8].

Among the many released signaling molecules, Hepatocyte Growth Factor (HGF) is a key player in the malignant crosstalk between the stroma and the primary tumor [9,10,11]. In malignant tumors, HGF is produced by stromal cells, while its receptor c-MET is expressed by cancer cells, which in the mid-1990s led to the hypothesis that this paracrine loop might determine malignant behaviors [12,13]. The involvement of HGF signaling, through its proto-oncogene receptor c-MET, is reported in the loss of epithelial phenotype and the acquisition of a migratory phenotype of non-cancerous cells as crucial event in the acquisition of a neoplastic phenotype, and it is widely documented in the progression of different types of cancer [12,14,15,16,17,18,19,20,21,22].

In particular, the role of HGF as mediator of the interactions between cancerous cells and adjacent stroma seems to be fundamental to create a microenvironment that promotes the further development and invasiveness of cancer [12,13,23]. HGF promotes the detachment of cancer cells from the primary tumor and their infiltration through the surrounding stroma favoring the pathways behind the degradation of the ECM [24]. HGF produced by stromal fibroblasts acts on cancer cells stimulating them not only to metastasize, but also to secrete HGF factor inducers by enhancing the connection between cancer cells and stroma mediated by HGF. These inductors are molecules involved in processes such as cell proliferation, angiogenesis and inflammation, mechanisms capable of modifying the tumor microenvironment and favoring tumor growth [9,13,25,26]. Thus, the mutual interactions between cancerous cells and fibroblasts, as mediated by HGF and HGF inducers, play a significant role in the occurrence of invasion and metastasis of the cancerous cells.

Here, we summarize the current knowledge about the different types of cells within the tumor microenvironment and their released factors that promote the metastatic outgrowth through the activation of ECM remodeling, inflammation, migration, angiogenesis, and invasion. This review aims to highlight the role of HGF in tumor formation, progression, and metastasis. We also dissect the potential use of new molecules that interfere with the HGF/c-MET axis as therapeutic targets for future clinical trials in cancer disease.

## 2. Cell Types and Signaling Molecules in Tumor Microenvironment

The tumor stroma mainly consists of the basement membrane, fibroblasts, ECM, immune cells, and vasculature. Although most host cells in the stroma possess certain tumor-suppressing abilities, the stroma changes during malignancy and eventually promotes tumor growth, invasion, and metastasis.

During early phases of tumorigenesis, the first cells that are recruited by a growing tumor mass are the fibroblasts as they represent the main constituents of the stromal tissue. These so-called cancer-associated fibroblasts (CAFs) have the fundamental role of secreting factors that act on the tumor cells in both paracrine and autocrine fashions resulting in a more aggressive cancer phenotype [7,27,28,29,30]. The main precursors of CAFs are normal fibroblasts, and the trans-differentiation of fibroblasts to CAFs is driven to a great extent by cancer-derived cytokines such as transforming growth factor-β (TGF-β). Across many cancer types, activated CAFs secrete growth factors, chemokines, collagens, and matrix-modifying enzymes, which collectively supply a communication network that governs cancer cell proliferation, tumor invasion, and metastasis across distant tissues. Within the tumor microenvironment, the tumor cells secrete several cytokines, which modulate the recruitment and function of the stromal cells. In particular, the tumor-derived RANTES/CCL5 cytokine stimulates CAFs to externalize the S100A4 protein which stimulates tumor cell survival and migration, up-regulation of the matrix metalloproteinases (MMPs), down-regulation of tissue inhibitors of the matrix metalloproteinases (MMPs) (TIMPs), activation of the transcription factor NF-κB and mitogen activated (MAP) kinase pathways, infiltration of T cells, and, finally, up-regulation of RANTES generating a signal amplification loop [31,32,33,34,35].

CAFs secrete monocyte chemotactic protein 1 (MCP1/CCL2) and stromal-cell-derived factor 1 (SDF1), also known as CXCL12, which are involved in the recruitment of myeloid-derived suppressor cells (MDSCs) and tumor-associated macrophages (TAMs) [36]. Tumor-derived stem cell factor (SCF) promotes the recruitment and activation of mast cells which induce proliferation, migration, and invasion of cancer cells, promote ECM degradation, induce angiogenesis, and recruit and modulate MDSC function [37,38]. At the tumor site, all of these immune cells secrete several chemokines and cytokines, which generate an inflammatory and immuno-suppressive state that critically contributes to malignant tumor progression.

**Figure 1:**
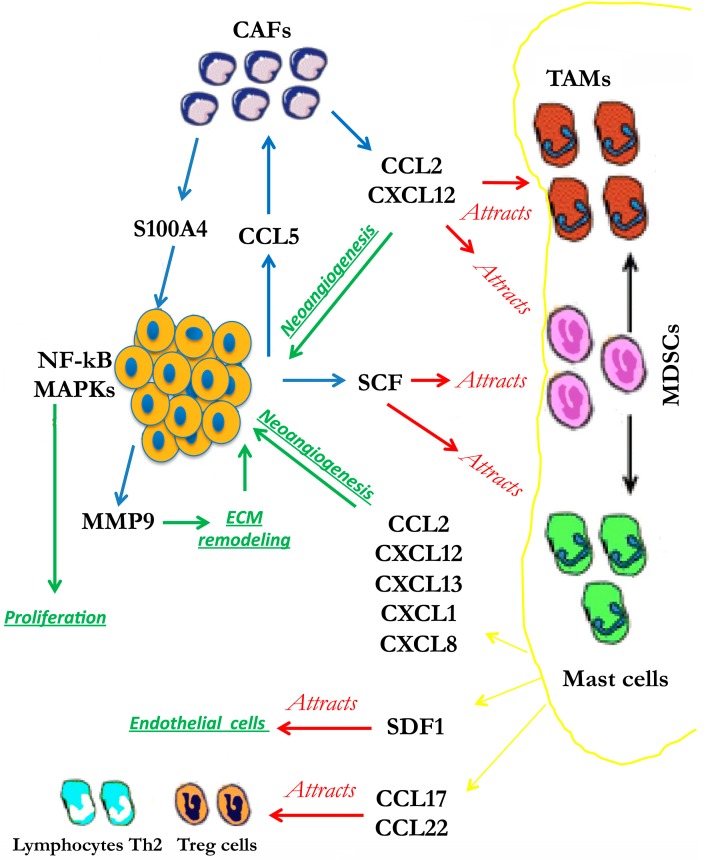
Formation of the tumor microenvironment.

Among other chemokines and cytokines secreted at tumor sites by immune cells, SDF1, a potent chemo-attractant for endothelial cells, the chemokines CCL2, CXCL8, CXCL1, CXCL13, and RANTES contribute to the neo-angiogenesis [36,39]. CCL17 and CCL22 preferentially attract T-cell subsets that are devoid of cytotoxic functions, such as regulatory T (Treg) cells and Th2 lymphocytes [40] (Figure 1).

**Figure 2:**
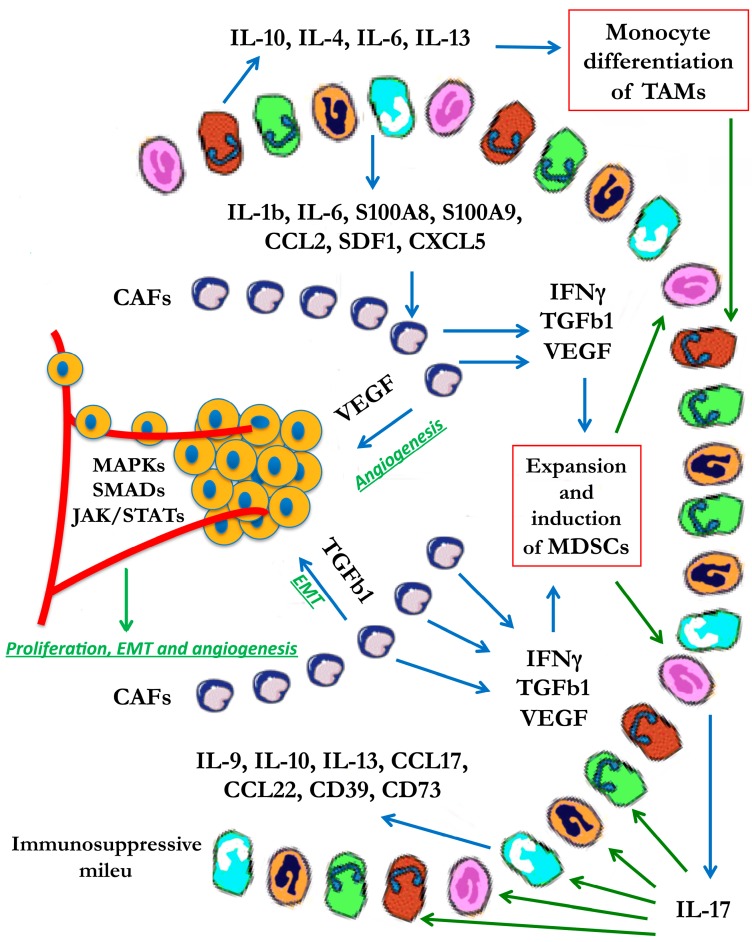
Inflammatory signaling in the tumor microenvironment.

Within the tumor microenvironment, the immunosuppressive milieu is further enhanced by the production of interleukin 10 (IL-10), which together with IL-4, IL-6, and IL-13 cytokines induces monocyte differentiation toward a mature M2-polarized phenotype that is characteristic of TAMs [36,41]. Moreover, at the tumor site, the IL-1 and IL-6 cytokines, the S100A8 and S100A9 pro-inflammatory proteins, the chemo-attractant molecules CCL2, SDF1 and CXCL5 are the main factors responsible for the recruitment and the induction of MDSCs [42,43]. The angiogenic vascular endothelial growth factor (VEGF) is one of the critical factors responsible for the expansion of MDSCs [44], while IL-4, IL-13, interferon (IFN)-γ, IL-1β, and TGF-β turn on their immunosuppressive functions [45,46]. In the tumor microenvironment, MDSCs produce high levels of IL-17, which further exacerbates the inflammatory tumor microenvironment. IL-17 leads to the up-regulation of IL-9, IL-10, IL-13, CCL17, CCL22, CD39 and CD73. This results in various actions: CCL17 and CCL22, in turn, are chemo-attractants that bring more Treg cells to the tumor sites; CD39 and CD73 enhance the suppressor functions of Treg cells [47,48]; IL-9 produced by Treg cells helps to maintain the survival of mast cells [49] which further contribute to the establishment of inflammatory and immuno-suppressive conditions at the tumor site by secretion of pro-inflammatory and immunosuppressive cytokines and chemokines. Finally, IL-10 and IL-13 induce MDSCs immunosuppressive functions [50] (Figure 2).

**Figure 3:**
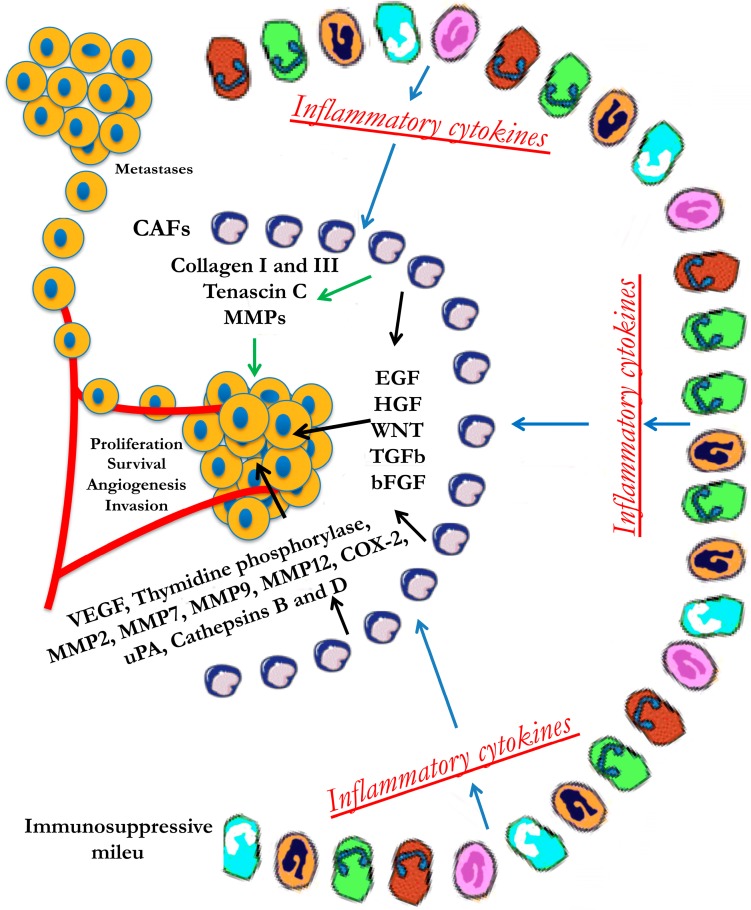
Invasive and metastatic signaling from the tumor microenvironment.

In the tumor microenvironment, the high levels of pro-inflammatory cytokines can induce actomyosin contractility in stromal fibroblasts, for example through signaling via the receptor subunit GP130-IL6ST, the tyrosine kinase JAK1, and Rho-kinase [51]. Stromal fibroblasts, in turn, remodel the ECM by secreting large amounts of collagen types I and III, tenascin C (TNC), and MMPs, and they create tracks for collective migration of cancer cells [52,53,54]. Hence, cytokine signaling pathways induce cell migration in both stroma and tumor cells. In conclusion, a consistent picture of how the stromal cells (CAFs and inflammatory cells, such as TAMs, MDSCs, and mast cells) can promote tumor malignant progression has been extensively depicted. Indeed, within the primary tumor microenvironment, the stromal cells provide potent oncogenic signals, such as TGF-β, HGF, epidermal growth factor (EGF), Wnt, and β-fibroblast growth factor (FGF), which stimulate cancer cell proliferation, survival, and invasion, thus facilitating metastasis. Moreover, these cells produce several angiogenesis-modulating enzymes, such as VEGF, thymidine phosphorylase, MMP2, MMP7, MMP9, MMP12, cyclooxygenase-2 (COX-2), urokinase plasminogen activator (uPA), and cathepsins B and D, which together degrade the ECM, thus again promoting metastasis (Figure 3). However, these mechanisms do not exclusively affect a single step of the metastatic cascade but are all highly pleiotropic.

## 3. HGF/c-MET Role within the Tumor Microenvironment

The receptor c-MET is primarily expressed in epithelial cells, while HGF is produced by surrounding mesenchymal cells. HGF/c-MET-mediated cross-talk between the epithelial and stromal compartments is required for normal physiological processes, and it is tightly regulated. Many tumors constitutively express both HGF and c-MET to evade spatial and temporal regulatory mechanisms. Activation of the HGF/c-MET axis induces different phenotypes depending on tumor stage: inducing proliferation and angiogenesis in primary tumors, stimulating motility to form micrometastases, and regaining the proliferation phenotype to form overt metastases [12,14,15,16,17,18,19,20,21,22].

*In vitro* studies conducted on liver tumor cells expressing c-MET co-cultured with CAFs from hepatocellular carcinoma (H-CAFs) demonstrated that HGF production was consistent with tumor volume growth, which led to the hypothesis that HGF/c-MET interaction plays a role in proliferation facilitated by H-CAFs [30]. HGF may also play a pivotal role in the regulatory circuit between gastric cancer cells and stromal fibroblasts, and neutralization of HGF inhibits both activation and tumor-promoting properties of CAFs [29]. A crosstalk between human adipose-derived mesenchymal stem cells (ADSCs) and breast cancer cells mediated by HGF/c-MET signaling has been reported to enhance tumor cells migration, acquiring a metastatic signature, and sustained tumor self-renewal [55]. ADSCs increase HGF production in presence of c-MET positive primary breast cancer cells, which in turn increase their HGF production; this observation confirms that the stroma creates a microenvironment where cancer cells are continuously stimulated to proliferate [56]. In addition to promoting cell proliferation of breast cancer cells, the paracrine HGF/c-MET signaling between fibroblasts and pre-invasive ductal carcinoma *in situ* cells (DCIS) enhances the transition to invasive carcinomas improving their ability to migrate, degrade collagen type IV, and to express and secrete uPA and uPAR [57]. Three-dimensional (3D) cultures of human mammary fibroblast (HMFs) result in an increased secretion of HGF compared to stromal fibroblasts cultured in 2D, subsequently enhancing the transition of DCIS to invasive ductal carcinoma (IDC) [58]. Finally, Wnt activity in colorectal cancer stem cells (CR-CSCs) has been described to be supported by myofibroblast-secreted HGF [59]. Indeed, the cytokines HGF, osteopontin, and stromal-derived factor 1α (SDF-1) secreted from tumor-associated cells increase CD44v6 expression in CR-CSCs by activating the Wnt/β-catenin pathway which promotes migration and metastasis [60,61].

Engagement of HGF with c-MET leads to activation of numerous signaling cascades, especially those related to invasion and properties of epithelial to mesenchymal transition (EMT) [19,20,22]. Among the signaling molecules activated are the non-receptors tyrosine kinases c-Src and c-Fyn [16]. In prostate cancer, Src is involved in cell growth at the metastatic site by affecting tumor invasion and bone turnover, whereas Fyn is involved in tropism of prostate cancer cells [21]. The c-MET receptor also interacts with CD44, integrins, and focal adhesion kinase (FAK) [15,62,63,64]. A CD44 isoform containing variant exon v6 sequences is strictly required for c-MET activation by HGF in rat and human carcinoma cells, in established cell lines as well as in primary keratinocytes [65]. HGF/c-MET binding up-regulates the expression of CD44v6 in murine melanoma cells through transcriptional activation of the immediate early gene *egr-1*; HGF seems to induce egr-1 activation via the Ras-Erk1/2 pathway [66]. c-MET is expressed or can be induced on immature, activated, and certain malignant B cells. HGF increases adhesion of c-MET positive B-lymphoma cells to fibronectin and collagen, mediated via beta 1-integrins, alpha 4 beta 1, and alpha 5 beta 1, and furthermore promotes migration and invasion [15]. In invasive and metastatic MTLn3 breast carcinoma cells, HGF stimulated both initial adhesion to and motility on the ECM ligands laminin 1, type I collagen, and fibronectin, and induced rapid tyrosine phosphorylation and activation of both c-MET and FAK [67]. Evidence indicate that the two signaling pathways, integrin/ECM and HGF/c-MET, cooperate synergistically to induce FAK activation in an adhesion-dependent manner, leading to enhanced cell adhesion and motility of tumor cells. It has also been shown that fibroblast-derived HGF triggers migration through the initial recruiting of integrins, cytoskeletal proteins, and FAK into focal adhesions in oral squamous carcinoma cells [68].

Other responses to HGF involve a sustained phosphorylation of Gab1 through Crk which contributes to the prominent activation of Rac1 leading to enhanced cell motility, scattering, and cell invasion of human synovial sarcoma cells [69]. Overexpression of HGF and c-MET has been shown to exert its effects in tumor progression in association with RhoA and probably with TIMP-3 in pulmonary non-small-cells which promotes EMT and carcinogenesis via up-regulation of COX-2 and Akt [19]. In the CT26 murine colorectal carcinoma cell line, the ERK/Akt pathway resulted to be particularly critical in the HGF-induced EMT process [22]. In mammary tumor cells, HGF/c-MET was found to regulate the signal transducer and activator of transcription 3 (Stat3) and MAP kinase signaling pathways by pharmacologic inhibition and small interfering RNA silencing, revealing a cooperative interaction between the two pathways to regulate HGF-induced invasion, scattering, and motility of mammary tumor cells [70]. Activated HGF/c-MET signaling is reported to play an important role in tumor stromal-interactions under hypoxia [71,72,73]. Hypoxic conditions induce a molecular response, in both normal and neoplastic cells, that drives the activation of a key transcription factor; the hypoxia-inducible factor-1 alpha (HIF-1α) [74]. Increased HIF-1α activates target genes involved in tumor cell growth, angiogenesis, metabolism, apoptosis, and metastasis [75]. HIF-1α through up regulation of HGF/c-MET signaling promotes cell migration towards the blood or lymphatic microcirculation. The stromal HGF expression was found to significantly correlate with not only the stromal HIF-1α expression but also the c-MET expression in pancreatic cancer cells PK8 [76].

**Figure 4:**
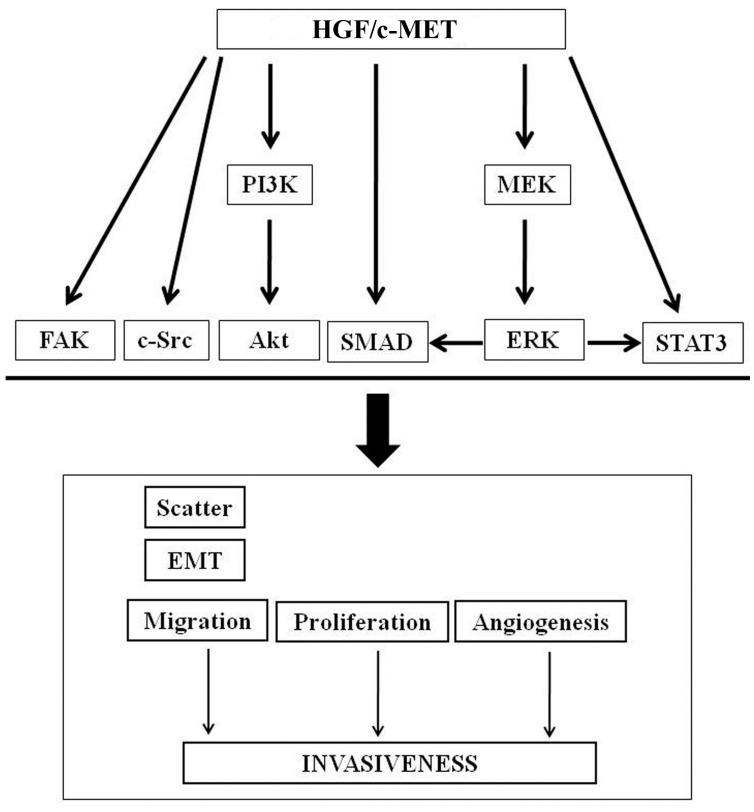
HGF/c-MET major signaling in tumor invasion.

The HGF/c-MET complex also increases the malignant potential of tumors through induction of VEGF-A production and suppression of thrombospondin-1, and acts synergistically with the VEGF receptor (VEGFR) through common downstream signaling molecules to increase neovascularization activity [77]. VEGF promotes angiogenesis and lymphangiogenesis in the primary tumor, providing the necessary routes for dissemination. VEGF-induced changes in vascular integrity and permeability promote both intravasation and extravasation, while VEGF-induced angiogenesis in the secondary tissue is essential for cell proliferation and establishment of metastatic lesions [75]. Overall, the interconnected and diverse functions of the HGF/c-MET axis in driving tumor growth support the role of the microenvironment milieu in directing the metastatic spread of the primary tumor (Figure 4).

## 4. Therapeutic Strategies Targeting HGF/c-MET Pathways

Among the currently available approaches for targeting HGF/c-MET pathways, there are small synthetic molecules, neutralizing monoclonal antibodies (mAbs) directed against HGF or c-MET, or HGF/c-MET competitors, many of which are in advanced stages of clinical trials [78,79,80,81,82,83].

The first group of compounds is represented by synthetic c-MET kinase inhibitors, small molecules capable to overcome the cellular membrane and inhibit cellular proliferation and/or angiogenesis preventing the mechanism of phosphorylation that is the start-point of these processes. Such molecules may competitively block the ATP-binding site in the catalytic domain of the c-MET receptor [84], as in the case of JNJ-38877605 and PF-04217903, or may be a non-ATP competitive inhibitor such as tivantinib (ARQ197) [85]. These inhibitors are specific for the c-MET receptor, but there are inhibitors capable of simultaneously inhibiting other receptors, such as crizotinib (PF-02341066), which also targets anaplastic lymphoma kinase [86], and foretinib (GSK-1363089/XL880), which also targets VEGFR2 [87].

The AMG 208 molecule selectively inhibits both ligand-dependent and ligand-independent c-MET activation, while golvatinib (E7050) targets both c-MET and VEGFR2 [88]. A combination strategy targeting both HGF/c-MET and VEGF has provided a novel therapeutic approach for treating patients with a broad spectrum of tumors [89,90,91,92]. The small molecule kinase inhibitor T-1840383 inhibits both HGF-induced c-MET phosphorylation and VEGF-induced VEGFR-2 phosphorylation in cancer epithelial cells and vascular endothelial cells, respectively. T-1840383 showed profound antitumor activity in a gastric tumor peritoneal dissemination model [90]. Cabozantinib, a tyrosine kinase inhibitor of c-MET and VEGFR-2 demonstrated clinical activity in patients with medullary thyroid cancer in phase I [91]. On the other hand, promising results were obtained by using a combination of the selective VEGFR inhibitor axitinib and the c-MET inhibitor, crizotinib in human renal cell carcinoma models [92]. Moreover, the therapeutic rationale of dual targeting of HGF/c-MET and other signaling pathways by small molecules is a current option for many cancers. A clinical study of MK8033, which targets c-MET and RON, is in progress [93]. Amuvatinib (MP470) inhibits PDGFR, c-Kit, and c-MET [94] in preclinical studies: MP470 combined with erlotinib inhibits prostate cancer cell proliferation and tumor xenograft growth [95], and MP470 treatment sensitizes glioblastoma cells to radiotherapy in mice [96].

Most of the mAbs prevent the pathways of signal transduction by directly binding c-MET and preventing the binding of HGF, resulting in increased apoptosis and decreased proliferation. Neutralizing mAbs against human HGF, such as L2G7, AMG102, and SCH900105 potently suppressed the growth of tumor xenografts in mice [97,98]. AMG102, currently in clinical trials, binds to the HGF light chain blocking HGF interaction with c-MET, and it is well tolerated in humans [99]. A humanized, bivalent anti-c-MET mAb, h224G11, inhibits c-MET phosphorylation and dimerization, and blocks proliferation, migration, invasion, morphogenesis, and angiogenesis in cell-based studies [100]. Another anti-c-MET mAb that blocks ligand binding, MetMab (ornatuzumab, formerly OA5D5), is an engineered monovalent antibody that has been shown to inhibit tumor growth in animal models by more than 95 percent [83]. MetMab downregulates constitutively active c-MET in tumor cell lines, and is currently in phase I/II human clinical trials in comparison with erlotinib in patients with non-small-cell lung cancer [101]. However, both approaches with competitive antagonists of HGF and mAbs are able to prevent the action of HGF on c-MET, but not to prevent its synthesis by fibroblasts. Recently published data has demonstrated that epigallocatechin-3-gallate (EGCG) is able to decrease HGF and VEGF serum levels in a phase II clinical trial in patients with prostate cancer as well as in CAFs *in vitro* [102]. This polyphenol appears to act at the level of ERK-mediated transcription to lower production of VEGF and HGF mRNA. HGF/c-MET competitors are decoys or antagonists that can inhibit the binding of HGF to the c-MET receptor competing with the ligand or receptor. An example is NK4, an HGF-like protein, which binds c-MET to saturate HGF binding sites, rendering the receptor inactive. Success with NK4 has been demonstrated both *in vitro* and *in vivo* [103].

Another unique approach has used inactive decoy c-MET receptors that prevent HGF interaction with both native c-MET and receptor dimerization. *In vivo* expression of decoy c-MET inhibits tumor cell proliferation and survival in a variety of human xenografts, impairs tumor angiogenesis by preventing host vessel arborization, and suppresses or prevents the formation of spontaneous metastases [104].

In addition to the compounds traditionally used to inhibit HGF/c-MET, new approaches include the use of miRNAs. Recent studies have demonstrated that some miRNAs regulate cancer metastasis production by modulating cancer cell-stroma interactions [105]. Several miRNAs have been identified which target the c-MET oncogene, including miR-34a, miR-199, miR-206, and miR-1 that could be challenged in therapies for silencing c-MET [106,107,108]. The miR-210 enhances mesenchymalstem cell survival in an oxidative stress environment through antioxidation and c-MET pathway activation [109]. HGF was identified as a target of miR-26a, a small non coding RNA involved in gene regulation of hepatocellular carcinoma (HCC): miR-26a down-regulation in HCC cells increases VEGF levels in the tumor microenvironment through the HGF/c-MET pathway, which induces the activation of VEGFR2 signaling in endothelial cells and promotes tumor angiogenesis in HCC [110].

The tumor microenvironment not only establishes the conditions for the growth and metastasis of the tumor, but also confers resistance to therapy [111]. Patients with BRAF-mutant melanoma show innate resistance to RAF inhibitors [112]. Immunohistochemistry experiments have shown a significant correlation between HGF expression by stromal cells and innate resistance to RAF inhibitor treatment. Inhibition of HGF or c-MET in combination with RAF inhibitor resulted in reversal of drug resistance, suggesting HGF or c-MET inhibitory agents as further potential therapeutic molecules to use in drug-resistance cases. Resistance to c-MET inhibition may be mediated through EGFR activation, or alternatively by increasing levels of c-MET amplification [83]. In order to optimize development of effective inhibitors of the HGF/c-MET pathway, clinical trials must be enriched for patients with demonstrable c-MET-pathway dysregulation for which robustly standardized and validated assays are required.

## 5. Conclusions

Significant progress has been made towards developing target-based cancer therapies over the past decade. Numerous molecules, designed to block specific signaling pathways important for tumor formation, progression, dissemination, and/or angiogenesis have been approved. Despite the remarkable success, most approved agents do not cure patients, and, in addition, some patients who initially respond to the treatments nevertheless develop resistance, and the tumors that emerge are often more aggressive and difficult to cure. Tumor cells have an extremely complex nature and are characterized by genetic heterogeneity and instability. On the other hand, tumor growth and progression are strongly influenced by specific activation responses of stromal cells near the growing tumor. Evidence indicates that even in tumors originated from the same tissue there are many differences that potentially contribute to the development of the disease. HGF and c-MET are key players in the tumor-stroma interactions, and HGF/c-MET signaling is strongly involved in tumor growth and progression, angiogenesis, and metastasis in many human cancers. Targeting HGF/c-MET signaling has provided valuable tools for the treatment of several malignancies and for overcoming drug resistance in cancer therapy. However, recent approaches demonstrate that the ultimate success in controlling most if not all cancers will possibly require the application of multiple agents that effectively inhibit different pro-cancer mechanisms.
